# Placenta-specific 8 limits IFNγ production by CD4 T cells *in vitro* and promotes establishment of influenza-specific CD8 T cells *in vivo*

**DOI:** 10.1371/journal.pone.0235706

**Published:** 2020-07-08

**Authors:** Chris D. Slade, Katie L. Reagin, Hari G. Lakshmanan, Kimberly D. Klonowski, Wendy T. Watford

**Affiliations:** 1 Department of Infectious Diseases, University of Georgia, Athens, GA, United States of America; 2 Department of Cellular Biology, University of Georgia, Athens, GA, United States of America; University of Iowa, UNITED STATES

## Abstract

During type 1 immune responses, CD4 T helper 1 (Th1) cells and CD8 T cells are activated via IL-12 and contribute to the elimination of intracellular pathogens through interferon gamma (IFNγ) production. In this study, we identified Placenta-specific 8 (Plac8) as a gene that is uniquely expressed in Th1 CD4 T cells relative to other CD4 T cell subsets and hypothesized that Plac8 may represent a novel therapeutic target in Th1 CD4 T cells. First, we determined that Plac8 mRNA in CD4 T cells was induced following IL-12 stimulation via an indirect route that required new protein synthesis. Upon evaluating the functional relevance of Plac8 expression in Th1 CD4 T cells, we discovered that Plac8 was important for suppressing IFNγ mRNA and protein production by CD4 T cells 24 hours after IL-12 stimulation, however Plac8 did not contribute to pathogenic CD4 T cell function during two models of intestinal inflammation. We also noted relatively high basal expression of Plac8 in CD8 T cells which could be further induced following IL-12 stimulation in CD8 T cells. Furthermore, Plac8 expression was important for establishing an optimal CD8 T cell response against influenza A virus via a T cell-intrinsic manner. Altogether, these results implicate Plac8 as a potential regulator of Th1 CD4 and CD8 T cell responses during Th1 T cell-driven inflammation.

## Introduction

Type 1 cell-mediated immune responses consist of CD4 T helper 1 (Th1) cells and CD8 cytotoxic T cells that provide protection against intracellular pathogens primarily through secretion of interferon gamma (IFNγ) and tumor necrosis factor (TNF) following IL-12 stimulation [1, 2]. Dysregulation of type I immune responses can lead to autoimmune disorders such as rheumatoid arthritis, multiple sclerosis, or inflammatory bowel disease [3]. Although some anti-inflammatory treatments for autoimmune diseases have shown promise, most leave the patients vulnerable to infections and require life-long treatment [4]. Before safer, more efficacious therapeutics against Th1-driven inflammation can be developed, a more complete understanding of the pathways that regulate this immune response is required.

Placenta-specific 8 (Plac8) is a small, cysteine-rich protein originally identified in placental tissue [5] but subsequently determined to be expressed in a variety of epithelial tissues and immune cells [6]. Currently, Plac8 is known to regulate oncogenic cell growth in various cancerous epithelial cell lines [7–12] and promote neutrophil antimicrobial functions [6]. However, Plac8 mRNA expression in T cells [6] suggests additional T cell-specific functions are likely. For example, Plac8 mRNA and protein is highly expressed in *Chlamydia muridarum*-specific Th1 CD4 T cells [13], Th1 CD4 T cells differentiated *in vitro* using classical Th1 biasing culture conditions [14], and more recently, in Th2 CD4 T cells isolated from mice sensitized and challenged with host dust mite allergen [15]. Altogether, these studies suggest that Plac8 may have an unappreciated role in promoting CD4 T cell differentiation and/or effector functions, conferring protective cellular immunity. A better understanding Plac8’s functional impact on Th1 CD4 T cells and other CD4 Th helper cell subsets is important for consideration of novel therapeutics and vaccines that may benefit from targeting this cellular pathway.

In this study, we show that Plac8 mRNA is differentially expressed by Th1 CD4 T cells compared to Th2, Th17, and iTreg T helper subsets differentiated *in vitro*. In addition, we determined that Plac8 expression is induced in CD4 T cells following Th1 stimulation with IL-12. Interestingly, Plac8 induction was via an indirect mechanism whereby IFNγ played a modest role. Parallel studies in naïve CD8 T cells revealed a surprising and, to date, an unreported high basal level of Plac8 mRNA expression relative to naïve CD4 T cells. Likewise, Plac8 expression is induced in CD8 T cells following IL-12 stimulation, suggesting a potential role for Plac8 in IL-12-driven Th1 immunity in both T cell subsets. Indeed, Plac8 suppressed IFNγ production by CD4, and to a lesser extent, CD8 T cells after IL-12 stimulation *in vitro*. Interestingly, however, *Plac8*^*-/-*^ CD4 T cells did not induce enhanced morbidity compared to *Plac8*^*+/+*^ CD4 T cells in a T cell transfer model of colitis in which Th1 cells are important mediators of disease. On the contrary, Plac8 was important for optimal establishment of virus-specific effector CD8 T cells in response to influenza infection. The numerical enhancement of CD8 T cells conferred by Plac8 sufficiency was T cell-intrinsic, yet unrelated to cellular proliferation and IL-2 signaling, both known to be important for effector cell differentiation. Although the precise function of Plac8 in T cells remains elusive, these data sufficiently demonstrate an immunoregulatory role for Plac8 during Th1 type immune responses that should be further explored for potential therapeutic benefit.

## Materials and methods

### Mice

Wild type (WT), C57BL/6 *IFNγ* ^*-/-*^, and C57BL/6 *Rag1*^*-/-*^ mice were originally ordered from the Jackson Laboratory or Charles River and are bred in-house. C57BL/6 *Plac8*^*-/-*^ mice were generated by Beverly H. Koller (University of North Carolina at Chapel Hill, Chapel Hill, NC) [6] and kindly provided by Dr. Raymond Johnson (Indiana University School of Medicine, Indianapolis, IN). C57BL/6-Tg (TcraTcrb)1100Mjb/J (OT-I) mice were originally provided by the late Dr. Leo Lefrançois (University of Connecticut, Farmington, CT), maintained on both CD45.1 or CD45.2 congenic backgrounds, and bred with *Plac8*^*-/-*^ mice to generate *Plac8*^*-/-*^, CD45.2^+^, OT-I mice. To generate bone marrow chimeras, WT CD45.1/.2 heterozygote mice were irradiated with a single dose of 1,100 rads. The following day, mice were injected i.v. with 3x10^6^ each WT CD45.1 and *Plac8*^*-/-*^ CD45.2 bone marrow cells and rested for 2 months for immune cell reconstitution. This study was carried out in strict accordance with the recommendations in the Guide for the Care and Use of Laboratory Animals of the National Institues of Health. The protocol was approved by the Institutional Animal Care and Use Committee at the University of Georgia (#A2018 06-013-A4). Euthanasia was performed by CO2 asphyxiation or by tribromoethanol overdose, both followed by cervical dislocation.

### Cell purification

Pooled spleens and lymph nodes were collected from WT or *Plac8*^*-/-*^ mice and processed by gently pressing them through a 70 μm filter. Where noted, CD4 and CD8 T cells were subsequently purified using a mouse T cell negative selection kit (Stemcell Technologies, Vancouver, Canada) according to manufacturer’s instructions. T cells were stained for 15 min at 4°C in PBS + 0.1% BSA (Gemini Bio-Products) using anti-mouse antibodies purchased from eBioscience and Tonbo Biosciences (both San Diego, CA): CD16/CD32 (93), CD4 (IM7), CD8 (53–6.7), CD44 (IM7), CD62L (MGL-14), CD45RB (C363.16A), and CD25 (PC61.5). Propidium iodide (Sigma-Aldrich) was used to exclude dead cells (PI^+^) from the sorted population. For T cell *in vitro* assays, live cells were designated as CD4^+^ or CD8^+^ T cells before sorting out naïve T cells (CD62L^+^, CD44^lo^) or memory T cells (CD44^hi^). For the T cell transfer model of colitis, live CD4^+^ T cells were sorted as (CD25^-^, CD45RB^hi^) effector cells.

### T cell culture

Naïve T cells were counted and plated at 1x10^6^ cells/mL on a 96 well culture plate coated with 5 μg/mL of α-CD3 (145-2C11) and α-CD28 (37.51) (Invitrogen, Carlsbad, CA). Cells were cultured for 3 days at 37°C in 5% CO_2_ and complete RPMI (RPMI 1640 containing 10% FBS, 100 U/ml penicillin, 100 μg/ml streptomycin, 2 mM L-glutamine, 0.01 M HEPES and 50 μM 2-mercaptoethanol). Differentiation conditions for CD4 T cells were: Th1 (10 ng/mL IL-12 + 5 μg/mL αIL-4), Th2 (10 ng/mL IL-4 + 5 μg/mL αIFNγ and αIL-12), Th17 (10 ng/mL IL-6 + 5 ng/mL TGF-β + 5 μg/mL αIL-2), or iTreg (10 ng/ml TFG-β + 40 IU/mL rhIL-2) (PeproTech, Rocky Hill, NJ and BD Sciences, San Jose, CA), or no additional cytokines (Th0) for the 3 days. Prior to IL-12 or IFNγ (PeproTech) stimulation, T cells were plated with 5 μg/mL of α-CD3 and α-CD28 for 3 days. Cyclohexamide (CHX) pretreatment was at 10 μg/mL for 30 min prior to assay where indicated. IFNγ protein from supernatants was measured using an IFNγ ELISA (eBioSciences).

### RT-qPCR

RNA was isolated from CD4 and CD8 T cells using an EZ-RNA extraction kit (Omega Bio-Tek, Norcross, GA) and was converted to cDNA using a high-capacity cDNA reverse transcription kit (Life Technologies, Carlsbad, CA). The relative expression levels of *plac8* and *ifnγ* and was determined by using predesigned TaqMan probe and primer sets relative to an actin endogenous control (Applied Biosystems, Grand Island, NY) and reference sample using the ΔΔC_T_ method.

### T cell transfer model of colitis

*Rag1*^*-/-*^ mice were injected i.p. with 3x10^5^ WT or *Plac8*^*-/-*^ naïve CD4^+^CD25^-^CD45RB^hi^ effector T cells. Mice were weighed prior to adoptive transfer and weekly thereafter until significant weight loss was observed in the WT control group. Splenic, mesenteric lymph node (mLN)-derived and lamina propria (LPL) lymphocytes were isolated. Cells were resuspended to 1x10^6^ cells/mL before *ex vivo* stimulation with 50 ng/mL PMA, 0.5 μg/mL ionomycin, and Golgi transport inhibitor according to the manufacturer’s directions (BD Biosciences) for 4 h at 37°C. Cells were surface stained for CD4 (RM4-5) and TCRβ (H57-597) prior to fixation in 4% formalin (Protocol), resuspension in BD Perm/Wash (San Jose, CA), and intracellular staining with monoclonal antibodies reactive to IFNγ (XMG1.2), TNF (MP6-XT22), and IL-17A (eBio1787). Samples were run on the BD LSRII flow cytometer, and data analysis was performed using FlowJo software.

### Influenza model

WT, *Plac8*^*-/-*^, or mixed bone marrow chimera mice were intranasally infected with 10^4^ pfu/mL influenza A/HK-x31 (X31, H3N2) or 10^3^ pfu/mL of recombinant X31 expressing ovalbumin (X31-OVA) in 50 μL PBS. OT-I adoptive transfers of 1x10^3^ WT and 1,000 *Plac8*^*-/-*^ OT-I cells were given i.v. one day prior to X31 infection. Mice were monitored until 8 or 10 dpi to quantitate the peak effector CD8 T cell response for X31-OVA or X31, respectively. To evaluate memory CD8 T cell responses, mice were euthanized at 35 dpi. BAL, mdLN, lung, and spleen were collected at indicated time points and processed as previously described [16]. Cells were stained with a combination of CD8 (53–6.7), CD44 (IM7), CD45.1 (A20), CD45.2 (104), TCR Vα2 (B20.1), CD25 (PC61.5), and the influenza nuclear protein (NP) MHC class-I [H-2Db/ASNENMETM] tetramer conjugated to brilliant violet 421 obtained from the NIH Tetramer Core Facility (Emory University, Atlanta, GA) at RT for 1 h. For proliferation studies, mice were administered 1 mg/100 μL of BrdU (Sigma) i.p. 12 hours prior to sacrifice, and single cell suspensions were intracellularly stained with an αBrdU (BU1/75) antibody. Plaque assays were done by serially diluting lung homogenates and plating them on MDCK cells as described [17].

### Statistics

*P* values were determined by unpaired or paired two-tailed Student’s t-test or by One-way ANOVA with Tukey’s post-hoc analysis using Prism software. Statistical significance is considered p ≤ 0.05.

## Results

### Plac8 mRNA is induced in T cells following IL-12 stimulation and suppresses IFNγ production by T cells in vitro

To first examine whether T cell development is fundamentally altered in *Plac8*^*-/-*^ mice, we compared T cell development in the thymi and spleens of WT and *Plac8*^*-/-*^ mice. T cell development in the thymus appeared completely normal in Plac8^-/-^ mice, with similar frequencies of DN1-4 populations, CD4^+^CD8^+^ double-positive (DP) and single-positive CD4 and CD8 T cells as WT mice (**S1A Fig**). Once they exit to the periphery a very minor, although statistically significant increase in the frequency of CD8 T cells was observed in the spleen, although this did not translate into an increase in the absolute number of CD8 T cells (**S1B Fig**). These data demonstrate that T cell development in *Plac8*^*-/-*^ mice is grossly normal.

**Fig 1:**
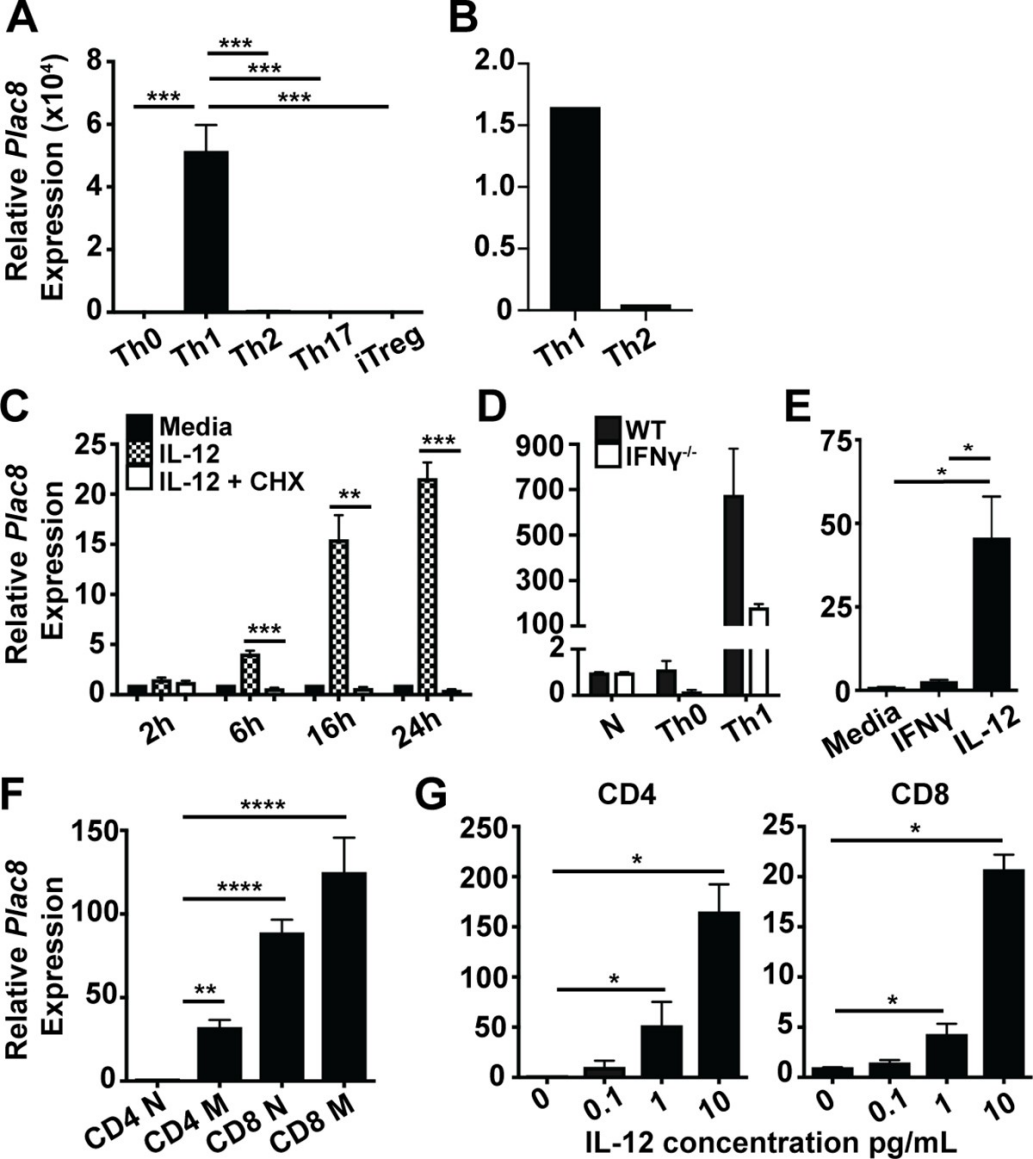
Plac8 mRNA is induced within T cells following IL-12 stimulation. (**A**) Naïve (CD44^lo^CD62L^+^) CD4 T cells were sort purified, differentiated over 3d into the indicated Th subsets *in vitro*, and the relative level of Plac8 mRNA expression determined. The level of Plac8 mRNA expression in undifferentiated Th0 CD4 T cells was set to 1 as the baseline expression level. (**B**) Plac8 expression in Th1 and Th2 *in vitro* differentiated CD4 T cells was determined by microarray [14]. (**C**) Naïve CD4 T cells were TCR activated with immobilized α-CD3 and α-CD28 for 3d prior to IL-12 stimulation for the indicated hours in the presence (white bars) or absence (hatched bars) of cycloheximide (CHX) pretreatment or media alone (filled bar). Plac8 expression was normalized to untreated Th0 CD4 T cells. (**D**) Naïve CD4 T cells were sort purified from WT and *IFN*γ^*-/-*^ mice, TCR activated in the presence or absence of IL-12 (Th1 and Th0, respectively) for 3d. Relative Plac8 expression levels were calculated using the naïve sample of each genotype as the baseline. (**E**) Naïve CD4 T cells were TCR activated and subsequently stimulated for 6h with IFNγ or IL-12 and Plac8 mRNA levels determined. (**F**) Naïve (N, CD44^lo^CD62L^+^) and memory (M, CD44^hi^) CD4^+^ and CD8^+^ T cells were sort purified from mice based on surface marker expression. Relative expression levels of Plac8 were normalized to CD4 N. (**G**) CD4 or CD8 T cells were TCR activated for 3d prior to stimulation with increasing concentrations of IL-12 for 24h. A, D, and F are representative of 3 experiments and C, E, and G are representative of 2 experiments. *P* values for C, D, F, and G were determined by unpaired Student’s *t* test. *P* values for A and E were calculated by one-way ANOVA and Tukey’s post hoc analysis. * (*P* < 0.05), ** (*P* < 0.01), *** (*P* < 0.001).

To date, Plac8 has been shown to be expressed by Th1 CD4 T cells stimulated with α-CD3 and α-CD28 in the presence of IL-12 *in vitro* [14] and by Th1 CD4 T present after *Chlamydia muridarum* infection *in vivo* [13]. However, to our surprise, a recent publication also noted high expression of Plac8 within Th2 cells [15]. To determine whether Plac8 expression is differentially regulated among CD4 T cell subset(s), we sought to globally assess Plac8 expression across a variety of *in vitro* differentiated CD4 T cell subsets. To this end, naïve CD4 T cells were sort-purified from mouse spleens and lymph nodes and plated with immobilized α-CD3 and α-CD28 supplemented with either IL-12 (Th1 conditions), IL-4 (Th2 conditions), TFG-β and IL-6 (Th17 conditions), TGF-β and IL-2 (iTreg conditions), or media alone (Th0) for three days [18] before being harvested and assessed for Plac8 mRNA expression via RT-qPCR. Differentiation of the specific CD4 T cell subsets was verified by assessing the expression of Th subset-specific signature cytokines or transcription factors by RT-PCR (**S2 Fig**). When the *in vitro* differentiated CD4 T cell samples were normalized to the level of Plac8 mRNA expression in undifferentiated Th0 cells, CD4 T cells cultured under Th1 conditions expressed significantly higher levels of Plac8 mRNA relative to all other subsets assayed (**Fig 1A**), consistent with the limited subset analysis by Wei et al. [14] (**Fig 1B**).

Because IL-12 is critical to the differentiation of Th1 CD4 T cells, we next determined whether IL-12 directly or indirectly induces Plac8 mRNA expression in Th1 cells. TCR-activated CD4 T cells were pretreated with or without cycloheximide (CHX) prior to IL-12 stimulation. CHX prevents new protein synthesis by interfering with protein translocation, thereby ceasing any protein intermediates produced upon IL-12 stimulation. Plac8 expression was significantly lower in CHX pre-treated CD4 T cells compared to their untreated counterparts, demonstrating that IL-12 induced Plac8 expression through a protein intermediate (**Fig 1C**). Given that IFNγ is a key product of the IL-12 signaling pathway and is important for further differentiating Th1 CD4 T cells [19], we examined whether Plac8 mRNA expression by Th1 CD4 T cells was dependent on IFNγ signaling. To test this, naïve CD4 T cells were isolated from C57BL/6 wild type (WT) mice and *IFNγ* ^*-/-*^ mice and cultured under Th1 conditions for three days, followed by quantitation of Plac8 mRNA expression. Although Plac8 mRNA induction after Th1 polarization trended lower in *IFNγ* ^*-/-*^ versus WT CD4 T cells, this was not statistically significant (**Fig 1D**). Likewise, although TCR-activated CD4 T cells stimulated directly with IFNγ for 6 h upregulated Plac8 mRNA expression, the level of expression observed was only a fraction of that observed in CD4 T cells stimulated with IL-12 (**Fig 1E**). Altogether, these data show that while IL-12 significantly induces Plac8 expression in CD4 T cells via a protein intermediate, IFNγ produced downstream of IL-12 plays only a modest role in Plac8 mRNA induction. Therefore, other unidentified protein intermediates downstream of IL-12 signaling contribute to Plac8 induction in CD4 T cells.

Because CD8 T cells also robustly respond to IL-12 stimulation, we next characterized Plac8 mRNA expression within these cells. First, naïve (CD62L^+^, CD44^lo^) and memory (CD44^hi^) phenotype CD8^+^ T cells, as well as CD4 T cells as a comparison, were isolated from spleen and lymph nodes, and the basal expression of Plac8 mRNA within these populations was quantified by RT-qPCR directly *ex vivo*. Naïve CD4 T cells expressed relatively low basal levels of Plac8 mRNA compared to other assayed cell types and thus were used as a reference point (**Fig 1F**). Plac8 mRNA expression was nearly 100-fold higher in naïve CD8 T cells relative to naïve CD4 T cells and there was about 30 times more Plac8 expression within memory CD4 T cells compared to naïve CD4 T cells (**Fig 1F**). To determine if IL-12 similarly induced Plac8 mRNA expression within CD8 T cells, naïve CD8 T cells were TCR-activated for three days prior to stimulation with increasing concentrations of IL-12 for 24 h. Like CD4 T cells, Plac8 mRNA expression within CD8 T cells was significantly induced in a concentration-dependent manner (**Fig 1G**). These data show that CD8 T cells express higher levels of Plac8 mRNA relative to CD4 T cells, and that Plac8 can be further induced in CD8 T cells following IL-12 stimulation, although the fold induction in the latter was significantly lower given their higher basal level of Plac8 expression.

**Fig 2:**
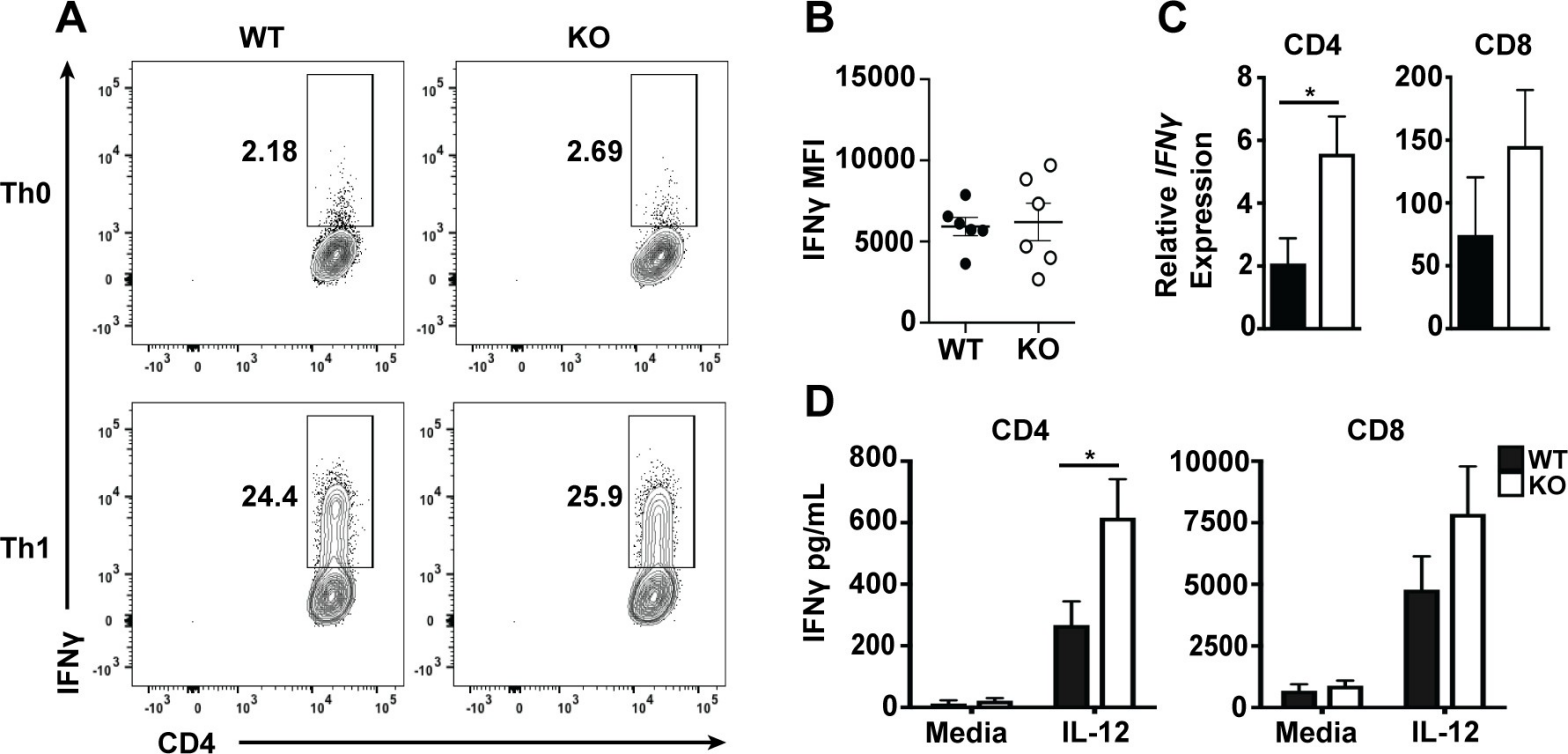
Plac8 negatively regulates IFNγ production by T cells following IL-12 stimulation. WT or *Plac8*^*-/-*^ (KO) naïve (CD44^lo^CD62L^+^) CD4 T cells were sort purified and TCR stimulated with α-CD3 and α-CD28 in the presence or absence of IL-12 (Th1 or Th0, respectively) for 3d (**A, B**). Cells were subsequently harvested, stimulated with PMA and ionomycin for 4h, and the frequency (**A**) of intracellular IFNγ levels and mean fluorescence intensity (MFI) (**B**) was determined by flow cytometry. Data are representative of two-independent experiments. Other WT or KO naïve CD4 T cells were TCR stimulated with α-CD3 and α-CD28 for 3d prior to IL-12 stimulation for 24h. Relative IFNγ mRNA levels (**C**) and protein levels (**D**) were determined by RT-qPCR or ELISA. Relative IFNγ expression was calculated using T cells that were not stimulated with IL-12 as the baseline. Each group has at least 6 samples. P values were determined by unpaired Student’s t test. * (P < 0.05).

Plac8 mRNA is preferentially expressed by Th1 versus other CD4 T cell subsets (**Fig 1A**). Therefore, it is possible that Plac8 plays an important role in either driving Th1 cell differentiation or promoting Th1 CD4 T cell effector function. To determine if Plac8 contributes to the differentiation of Th1 CD4 T cells, naïve WT and *Plac8*^*-/-*^ CD4 T cells were isolated and differentiated in Th1 conditions for three days, and polarization was subsequently assessed by measuring intracellular IFNγ staining by flow cytometry. Overall, there was no difference in either the frequency of IFNγ-producing CD4 T cells or IFNγ mean fluorescence intensity between WT and Plac8^-/-^ Th1 cells (**Fig 2A and 2B)**, suggesting that Plac8 does not contribute to Th1 differentiation. To determine if Plac8 contributes to differences in T cell IFNγ production, WT and *Plac8*^*-/-*^ CD4 or CD8 T cells were TCR-activated with α-CD3 and α-CD28 for three days prior to IL-12 stimulation for 24 h. After IL-12 stimulation, supernatants were collected, and the mRNA isolated from cell pellets was converted into cDNA for analysis of IFNγ gene expression by RT-qPCR. *Plac8*^*-/-*^ CD4 T cells stimulated with IL-12 had higher levels of IFNγ mRNA compared to WT CD4 T cells and the same trend was observed in CD8 T cells (**Fig 2C**), suggesting that Plac8 may negatively regulate IFNγ transcription. Indeed, *Plac8*^*-/-*^ CD4 T cells consistently secreted higher levels of IFNγ protein as determined by ELISA (**Fig 2D**). These data suggest that although Plac8 is not essential for Th1 cell differentiation, Plac8 constrains IFNγ production by CD4 T cells early after IL-12 stimulation.

**Fig 3:**
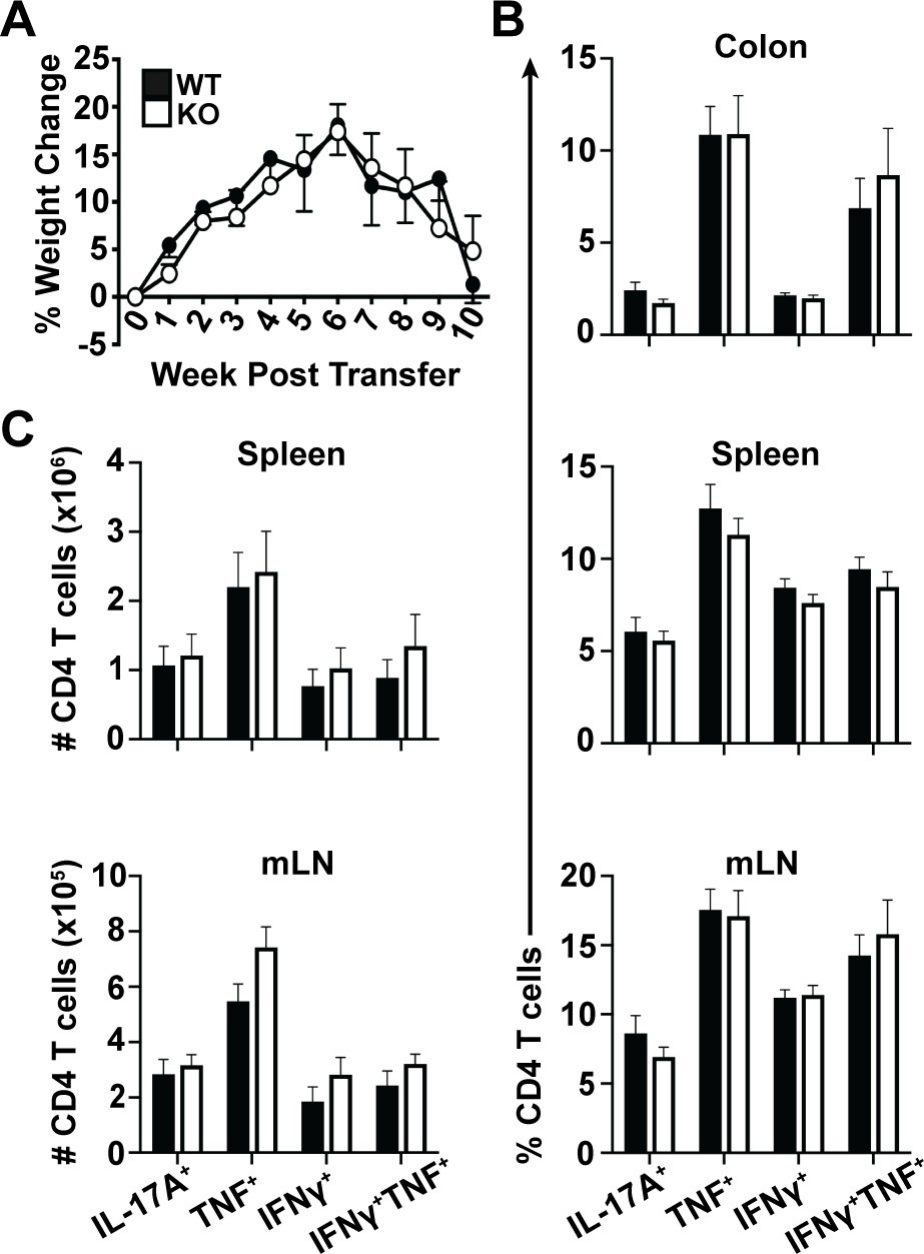
Plac8 does not suppress IFNγ production by pathogenic CD4 T cells during colitis. WT or *Plac8*^*-/-*^ (KO) CD4^+^CD45RB^hi^CD25^-^ effector T cells were injected into *Rag1*^*-/-*^ recipients. (**A**) Mice were weighed weekly and % weight change was calculated. Data shown are the daily mean % weight change for each group and representative of three independent experiments and 6 mice per group. After colitis onset, mice were sacrificed and single cells were isolated from the indicated tissues and stimulated with PMA and ionomycin for 4h. The frequency (**B**) and total cell number (**C**) of the indicated cytokine-producing CD4 T cells was determined by intracellular flow cytometry. B and C are pooled data from 3 independent cohorts totaling 17 hosts/group.

Given that Plac8 inhibits IFNγ production by CD4 T cells *in vitro*, we sought to evaluate whether Plac8 functionally impairs Th1 CD4 T cells *in vivo* by utilizing a well-established T cell transfer model of colitis in which adoptively transferred CD4^+^CD45RB^hi^CD25^-^ T cells induce colonic inflammation in *Rag*^*-/-*^ recipients within 8–12 weeks post-transfer [20]. Inflammation in this colitis model is driven by the recognition of gut flora by self-reactive T cells through the IL-23/IFNγ/IL-17 axis [21]. IFNγ produced by Th1 cells in this model is known to be critical for disease pathogenesis, because transfer of *IFNγ* ^*-/-*^ CD4 T effector cells fails to induce disease in *Rag1*^*-/-*^ hosts [22]. In addition, since development of disease is completely dependent on the transferred CD4 T cell population, this model will allow us to evaluate the direct effect of Plac8 on pro-inflammatory CD4 T cell function *in vivo*. Therefore, to determine if Plac8 contributes to IFNγ suppression *in vivo*, *Rag1*^*-/-*^ recipients received either WT or *Plac8*^*-/-*^ CD4^+^CD45RB^hi^CD25^-^ T cells, and morbidity was assessed via weight loss over time. There was no difference in weight loss between the two groups of animals at any time point analyzed (**Fig 3A**). Ten weeks post-transfer, CD4 T cells were evaluated for TNF, IL-17A, and IFNγ cytokine production via intracellular cytokine staining; however, there was no difference in either the frequency (**Fig 3B**) or total cell number (**Fig 3C**) of cytokine-producing WT and *Plac8*^*-/-*^ cells in the spleen, mesenteric lymph nodes (mLN), or colon. Because we saw an increase in CD4 T cell IFNγ production 24 h-post IL-12 stimulation *in vitro*, and the T cell transfer model of colitis induces chronic inflammation over several weeks, we hypothesized that Plac8’s impact on CD4 T cell IFNγ may be limited to models of acute inflammation. To this end, we utilized an acute infectious model of colitis in response to *Citrobacter rodentium*. This pathogen elicits a potent Th1 and Th17 response; however, IFNγ production by CD4 T cells is indispensable [23]. To specifically address the T cell-intrinsic regulation of the response, we utilized mixed bone marrow chimeras whereby irradiated CD45.2/.1 heterozygote hosts were reconstituted with 1:1 ratio of WT (CD45.1) and *Plac8*^*-/-*^ (CD45.2) bone marrow. Because the WT and *Plac8*^*-/-*^ T cells are exposed to the same environmental stimuli in this model, any differences noted between the two cell types are due to intrinsic differences within the T cells themselves. However, assessment of cytokine production by WT and *Plac8*^*-/-*^ CD4 T cells 14 days post *C*. *rodentium* infection revealed no differences in TNF, IL-17A, or IFNγ production (**S3 Fig)**. Therefore, while our *in vitro* stimulation data suggested that Plac8 may function to constrain IFNγ production, our functional analyses suggests that Plac8 does not contribute to T cell-mediated inflammation *in vivo*, at least in the specific Th1-dependent disease models tested.

### Plac8^-/-^ hosts establish fewer memory CD8 T cells after influenza infection

**Fig 4:**
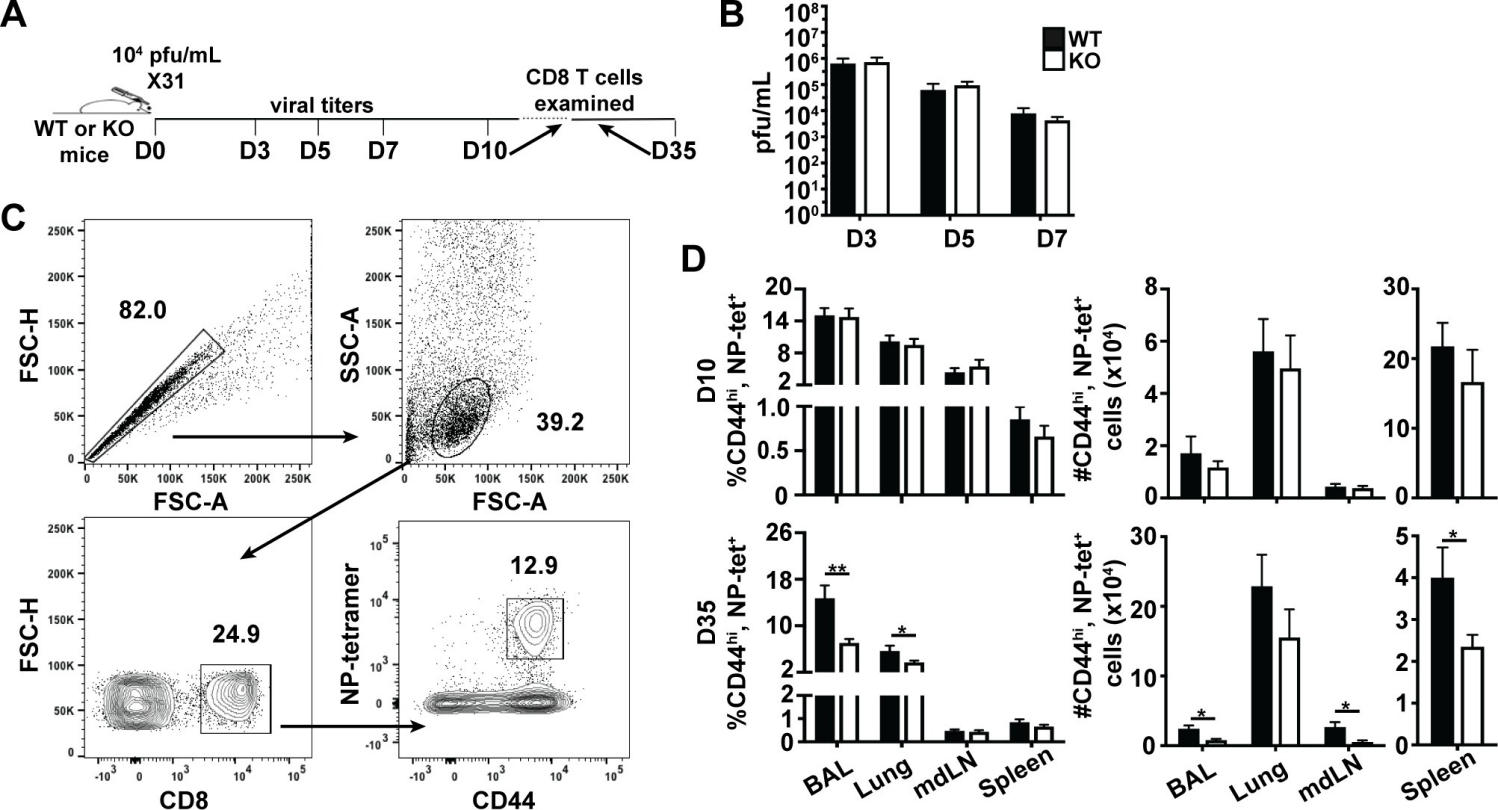
Plac8^-/-^ hosts establish fewer memory CD8 T cells after influenza infection. (**A**) WT or *Plac8*^*-/-*^ (KO) mice were intranasally infected with 10^4^ pfu/mL of X31. (**B**) On 3, 5, and 7 dpi, mouse lungs were harvested and viral titers determined via plaque assays. N ≥ 6 mice/ time point. (**C**) Gating strategy used to identify influenza NP-specific CD8 T cells by flow cytometry. (**D**) The frequency and total cell number of NP-specific CD8 T cells isolated from the indicated tissues at 10 or 35 dpi. Data represents 12 animals (3 pooled cohorts) or 17 animals (four pooled cohorts) for 10 and 35 dpi, respectively. *P* values were determined by unpaired Student’s *t* test. * (*P* < 0.05), ** (*P* < 0.01).

Because CD8 T cells express relatively high basal levels of Plac8 mRNA (**Fig 1F**), and there were trending differences in IFNγ regulation between WT and *Plac8*^*-/-*^ CD8 T cells stimulated with IL-12 (**Fig 2C and 2D**), we hypothesized that Plac8 may contribute to CD8 T cell function *in vivo*. To test this, we utilized a mouse-adapted influenza A virus (IAV) infection model, because IAV induces a robust Th1 immune response in C57BL/6 WT mice [24], and CD8 T cells play a critical role in viral clearance [25]. First, we determined whether Plac8 contributed to viral clearance by assessing lung viral titers from WT or *Plac8*^*-/-*^ mice infected with 10^4^ pfu of X31 (**Fig 4A**); however, there were no differences in viral titers on 3, 5, or 7 days post infection (dpi) between WT and *Plac8*^*-/-*^ mice (**Fig 4B**) indicating that Plac8 does not significantly impact early CD8 T cell viral control.

In other cell types, Plac8 is known to regulate cellular proliferation [12, 26, 27], and modified proliferation can impact CD8 T cells not only acutely during the effector phase of the anti-viral response, but also the development of subsequent memory CD8 T cells. Therefore, we next assessed whether Plac8 is important for the establishment of the immunodominant influenza nucleoprotein (NP)-specific CD8 T cells within WT and *Plac8*^*-/-*^ mice. WT and *Plac8*^*-/-*^ mice were infected with 10^4^ X31, and NP-reactive CD8 T cells were examined in the bronchoalveolar lavage fluid (BAL) as well as in lung, spleen and lung-draining mediastinal lymph node (mdLN) tissues at the peak of the effector (10 days post infection, dpi) and memory (35 dpi) CD8 T cell response (**Fig 4A & 4C**). At 10 dpi, all tissues assessed harbored an equivalent frequency and number of NP-specific CD8 effector T cells (**Fig 4D**). However, there was a significantly lower frequency and number of NP-specific memory CD8 T cells in the *Plac8*^*-/-*^ IAV infected mice compared to WT mice at the 35 dpi memory time point. These data suggest that Plac8 is important for the establishment of memory CD8 T cells.

### Plac8 promotes effector CD8 T cell establishment through a T cell-intrinsic mechanism that is not proliferation

**Fig 5:**
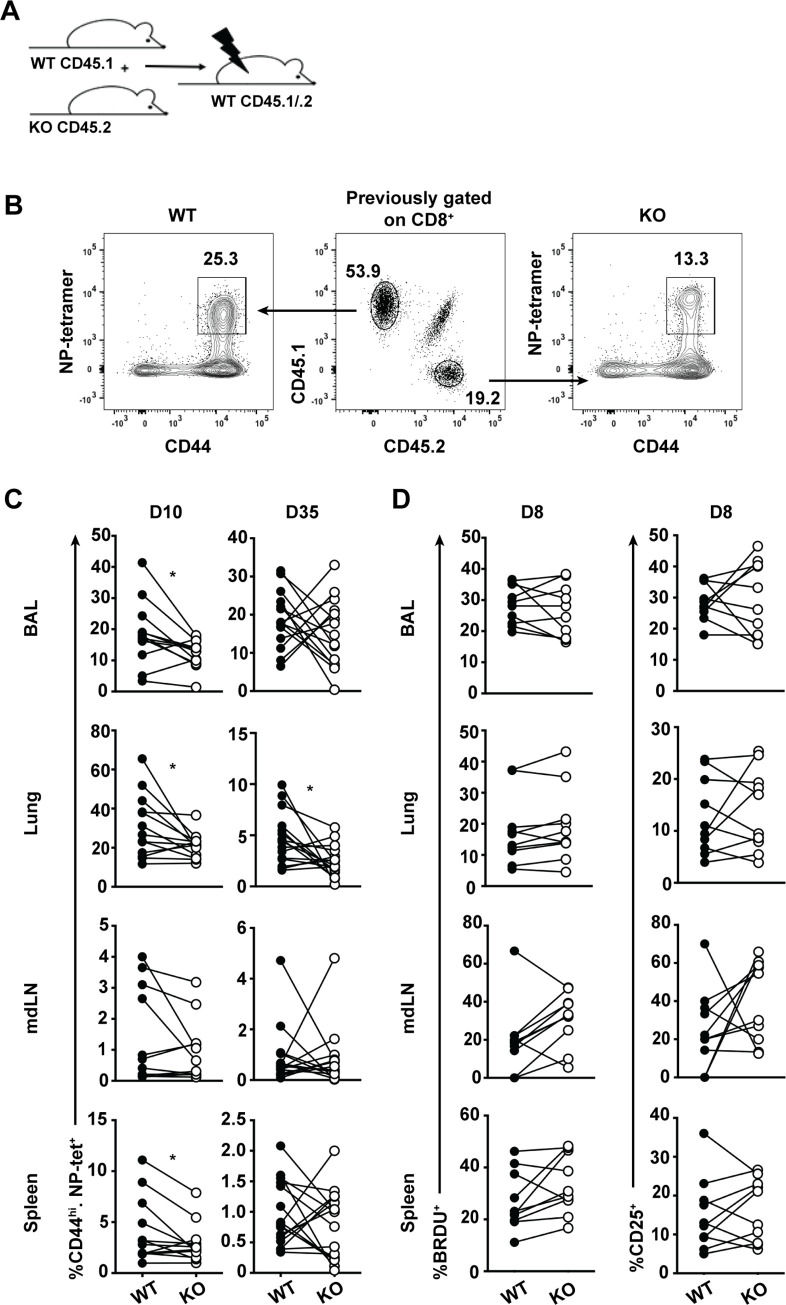
Plac8 promotes effector CD8 T cell establishment through a T cell-intrinsic mechanism, independent of proliferation. (**A**) Mixed bone marrow chimeras were generated by injecting WT CD45.1 and KO CD45.2 bone marrow 1:1 into irradiated CD45.1/.2 recipients and resting for 2 months for reconstitution. (**B**) Gating strategy to identify the genotype of influenza NP-specific CD44^hi^ CD8 T cells responding to infection based on CD45 status. (**C**) Chimeras were infected with influenza X31 and the frequency of NP-specific CD8 T cells of derived from WT or KO bone marrow was determined 10 (n = 13) and 35 (n = 16) dpi using the gating strategy in (**B**). Lines connect the frequency of WT and KO representation in a single host and the data are pooled from 3 independent experiments. (**D**) Mice were infected as in (**C**) and subsequently injected with BrdU i.p. 12h before a harvesting the tissues 8 dpi. Data depict BrdU incorporation within CD44^hi^, NP-tetramer^+^ CD8 T cells over the 12h labelling period and CD25 expression by these cells (**E**) in 2 pooled independent cohorts of mice (n = 11). P values were determined using paired Student’s t test. * (P < 0.05).

Plac8 protein is expressed by other immune and stromal cells, such as macrophages and lung epithelial cells [6], that participate in the immune response to influenza, and can indirectly influence the influenza-specific CD8 T cell response. Therefore, we cannot differentiate if the phenotype observed at 35 dpi (**Fig 4D**) is the result of a CD8 T cell intrinsic or extrinsic mechanism. To circumvent this limitation, we utilized a mixed bone marrow chimera model whereby CD45.1/.2 WT recipient mice were lethally irradiated and reconstituted 1:1 with bone marrow from CD45.2 *Plac8*^*-/-*^ and CD45.1 WT mice (**Fig 5A**). After a two-month engraftment period, chimeric mice were infected with 10^4^ pfu of X31, and BAL, spleen, mdLN, and lungs were harvested 10 or 35 dpi. Although each recipient mouse was given a 1:1 mix of bone marrow, not all hosts engrafted equally. Therefore, to negate any engraftment variability, CD8^+^ T cells derived from WT (CD45.2^+^) and *Plac8*^*-/-*^ (CD45.1^+^) mice were identified, and the frequency of CD44^hi^, NP-reactive cells from each genotype was quantitated **(Fig 5B)**. At 10 dpi, *Plac8*^*-/-*^ CD44^hi^, NP-specific CD8 T cells were present at a lower frequency than their WT NP-specific counterparts in the lung, BAL and spleen, whereas memory NP-specific CD8 T cells derived from *Plac8*^*-/-*^ mice were additionally reduced in the lung (**Fig 5C and 5D**). These data suggest that Plac8 is regulating the CD8 T cell response through a T cell-intrinsic mechanism.

To examine whether the phenotype observed at the effector time point could be explained by differences in CD8 T cell proliferation, another cohort of chimeric mice were infected with X31, and the proliferation of NP-specific CD8 T cells were characterized 8 dpi, just prior to the peak proliferative CD8 T cell response, via 5-Bromo-2’-deoxyuridine (BrdU) incorporation. Simultaneously, surface expression of CD25, the high affinity IL-2Rα chain, was measured to evaluate potential differences in cellular responsiveness to the IL-2 growth factor. However, no differences in either CD25 surface expression or BrdU incorporation were observed at the assayed time (**Fig 5E**). Altogether, these data demonstrate that Plac8 is important for the establishment of effector CD8 T cells 10 dpi, independent of modified proliferation or IL-2 signaling.

**Fig 6:**
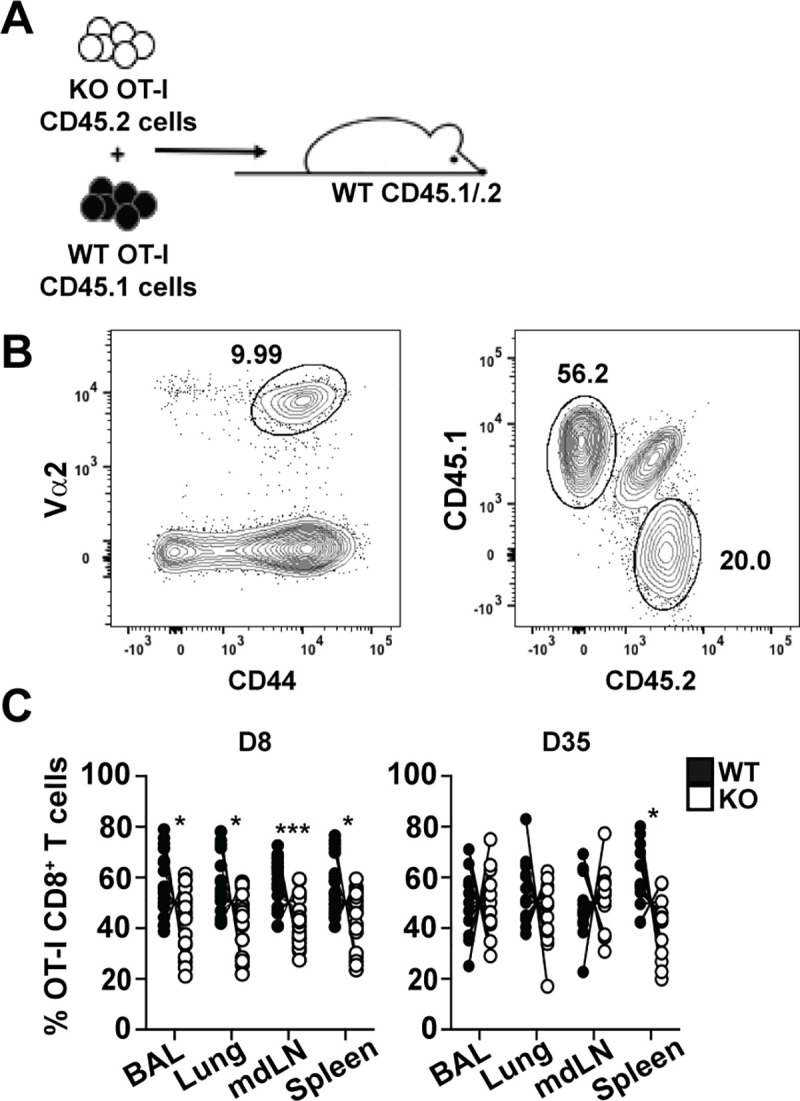
Plac8’s impact on effector CD8 T cell establishment is unrelated to differences in NP-specific precursors. (**A**) Mice received a 1:1 mix of WT (CD45.1) and *Plac8*^*-/-*^ (CD45.2) OT-I cells one day prior to infection with 10^3^ X31-OVA. (**B**) OT-I cells were identified as CD44^hi^ and TCR Va2^+^ CD8 T cells and genotyped based on CD45 status. (**C**) BAL, lung, mdLN, and spleen were harvested 8 dpi and 35 dpi and the frequency of WT vs Plac8^-/-^ OT-I cells participating in the response to infection was determined. Lines connect the frequency of the two donor OT-I populations within a single recipient. *P* values were determined by paired Student’s *t* test. * (*P* < 0.05), *** (*P* < 0.001).

Given that WT and *Plac8*^*-/-*^ mice could harbor different numbers of NP-specific CD8 T cell precursors, which could directly impact the magnitude of the NP-specific CD8 T cell response, we repeated the influenza studies using an OT-I T cell adoptive transfer model whereby all transgenic OT-I cells (WT or *Plac8*^*-/-*^) are TCR Vα2^+^ CD8^+^ T cells that recognize a specific ovalbumin (OVA) peptide in the context of MHC class I. WT CD45.1/.2 recipient mice received 1,000 WT (CD45.1^+^) and 1,000 *Plac8*^*-/-*^ (CD45.2^+^) OT-I cells one day prior to infection with a recombinant X31 virus expressing the OVA antigen (X31-OVA) (**Fig 6A**). BAL, spleen, mdLN, and lung were harvested at the peak of the OT-I cell response and at a memory time point (8 and 35 dpi, respectively), and OT-I cells were identified as CD8^+^, CD44^hi^, and TCR Vα2^+^ cells before distinguishing a CD45.1^+^ (WT) or CD45.2^+^ (*Plac8*^*-/-*^) origin (**Fig 6B**). There were significantly less *Plac8*^*-/-*^ OT-I cells in all analyzed tissues at 8 dpi, and significantly less *Plac8*^*-/-*^ OT-I cells in the spleen at 35 dpi (**Fig 6C**). Notably, the *Plac8*^*-/-*^ influenza-specific CD8 T cells that were present produced similar levels of IFNγ as their WT counterparts (**S4A and S4B Fig**), consistent with the *in vitro* data showing similar IFNγ production by both WT and *Plac8*^*-/-*^ CD8 T cells (**Fig 2C and 2D**). Because the starting amount of adoptively transferred WT and *Plac8*^*-/-*^ OT-I cells are equal, and there are significantly fewer *Plac8*^*-/-*^ OT-I T cells at the peak of the T cell response, these observations provide strong evidence that Plac8 promotes effector CD8 T cell establishment through a T cell-intrinsic mechanism.

## Discussion

In this study, we have determined that Plac8 is highly and uniquely expressed by Th1 CD4 T cells compared to Th2, Th17, and iTreg CD4 T cells. We also found that Plac8 is indirectly induced following IL-12 stimulation in CD4 T cells and that IFNγ plays a modest role in its induction. While Plac8 significantly suppressed IFNγ production in IL-12 stimulated CD4 T cells *in vitro*, this phenotype was not captured in either a T cell transfer model of colitis or following *Citrobacter rodentium* infection. Interestingly, and unexpectedly, we found that naïve CD8 T cells express significantly higher basal levels of Plac8 compared to naïve CD4 T cells (~100x greater) and that IL-12 similarly induces Plac8 mRNA expression within CD8 T cells. Further, the functional effect of Plac8 deficiency was more relevant to CD8 T cell responses as *Plac8*^*-/-*^ mice harbored fewer effector and, in some cases, memory antigen-specific CD8 T cells after IAV infection.

Determining factors that regulate Plac8 expression in T cells may help to identify how it can be utilized therapeutically during T cell-driven inflammation. Data mined from Wei *et al*. showed that Plac8 mRNA expression was high in Th1 CD4 T cells relative to Th2 CD4 T cells differentiated *in vitro* [14] and Johnson *et al*. found that Plac8 mRNA and protein expression is associated with Th1 *Chlamydia muridarum*-specific CD4 T cells [13]. However, more recently, Tibbitt *et al*. identified Plac8 as one of the most highly upregulated genes in Th2 CD4 T cells isolated from mice sensitized and challenged with house dust mite (HDM) allergen *in vivo* [15]. These seemingly contradictory results might be due to variations in the differentiation conditions found *in vivo* versus *in vitro*. For example, IL-1α and TSLP, which are not typically included in *in vitro* cultures, including ours, are secreted during HDM sensitization [28] and their impact on Plac8 expression in CD4 T cells has not been determined. Altogether, these results indicate that Plac8 induction in CD4 T cells may be complex and context dependent, and a careful evaluation of the environments that induce Plac8 expression in CD4 T cells will be important as we continue ascribing Plac8’s function in these cells.

Our *in vitro* studies firmly identify IL-12 as a factor that increases Plac8 mRNA expression in CD4, and to a lesser extent, CD8 T cells. Since our data demonstrate that IL-12 induces Plac8 expression *indirectly* (**Fig 1C**), and the fact that Plac8 is referred to as an IFN-stimulated gene [29, 30], we initially hypothesized that autocrine IFNγ signaling, secondary to IL-12, was responsible for the significant Plac8 expression observed in Th1 CD4 T cells. However, direct stimulation of T cells with IFNγ failed to induce Plac8 expression to a similar extent as IL-12, suggesting that IL-12 signaling induces Plac8 mRNA expression indirectly via a largely IFNγ-independent mechanism. Genomic analysis of 1kb upstream of Plac8’s promoter sequence identified putative binding sites for C/EBPα, C/EBPβ, NF-κB, Sp1, and IRF8, but not Stat4 which is directly regulated by IL-12 [31]. C/EBPα is the only one of these transcription factors implicated in limiting IFNγ production by Th1 CD4 T cells through a T cell-intrinsic manner [32], consistent with the phenotype we observed in our Plac8 study. This suggests that C/EBPα could be regulating Plac8 expression in response to IL-12 stimulation; however, it remains unclear whether IL-12 can *directly* regulate C/EBPα production in CD4 T cells. It should be noted that we were unable to directly assess the regulation of Plac8 protein levels due the absence of a murine-reactive Plac8 antibody. For future studies, it will be important to generate a mouse-reactive Plac8 antibody that will enable protein quantitation by Western blot to determine how efficiently Plac8 mRNA is translated into protein in various cell lineages and to validate high Plac8 *protein* expression within the Th1 lineage.

We determined that Plac8 suppresses IL-12-induced IFNγ production by CD4 T cells at the transcriptional level (**Fig 2C**). While these findings were initially unexpected, a previous study demonstrated that Plac8 is important for brown fat differentiation through associating with C/EBPβ and binding to the C/EBPβ promoter to induce its transcription [33], so the possibility that Plac8 could function as a transcriptional regulator is not unprecedented. Despite Plac8’s regulation of IFNγ production by CD4 T cells *in vitro*, Plac8 ablation did not apparently alter T cell effector function in a T cell transfer model of colitis (**Fig 3**). This may be because IL-12p70 is known to be dispensable for colitis, but IL-23 and IFNγ are not [23, 34], suggesting that IFNγ production by CD4 T cells may result from IL-23 stimulation rather than IL-12 stimulation in this model. It is also possible that Plac8’s effect on CD4 T cell IFNγ production occurs transiently, and the magnitude of the enhancement of the IFNγ response in *Plac8*^*-/-*^ T cells may not be large enough to manifest during a model of chronic inflammation such as this colitis model.

Surprisingly, Plac8 was important for the establishment of effector influenza-specific CD8 T cells through a T cell-intrinsic manner when utilizing a mixed bone marrow chimera model and an OT-I T cell adoptive transfer model (**Figs 5C and 6C**). However, when comparing the NP-specific CD8 T cell response in the complete WT versus *Plac8*^*-/-*^ influenza mouse model, Plac8 was important for the establishment of memory, but not effector, CD8 T cells (**Fig 4E**). Because the impact of Plac8 on the influenza-specific CD8 T cell response is different when utilizing a model where Plac8 is globally depleted versus a model where Plac8 is specifically depleted in CD8 T cells, this data suggests that Plac8 may contribute to the function of other immune cell(s) during influenza infection. Plac8 protein is expressed by macrophages and lung epithelial cells, two key components during IAV infection [6], but there is no information regarding Plac8’s function within these cells. Therefore, it will be important to determine Plac8’s impact on global immune cell function and CD8 T cell-specific functions during influenza infection.

When Plac8 was depleted in CD8 T cells, we observed a decrease in *effector* CD8 T cell establishment relative to WT CD8 T cells which led us to hypothesize that Plac8 promotes proliferation within CD8 T cells. This hypothesis is supported by Plac8’s documented role in promoting proliferation in multiple cancerous epithelial cell lines [12, 26, 27]. However, in our model we found that Plac8 does not regulate the proliferation of antigen-specific CD8 T cells as measured by similar surface expression of CD25 and BrdU incorporation 8 dpi (**Fig 5E**). We also compared the WT and KO effector CD8 T cells based on their expression of surface markers IL-7Rα and KLRG1 which are indicative of effector CD8 T cell fate, however there were no differences in the expression of these surface markers, suggesting that Plac8 does not impact CD8 T cell differentiation pathways during influenza infection (**S4C and S4D Fig**). An alternate hypothesis is that Plac8 may promote CD8 T cell survival after IAV infection because it has been shown to promote cancer cell survival by limiting apoptosis [35, 36]. Because apoptotic cells are rapidly scavenged *in vivo* by phagocytes, quantifying apoptosis *in vivo* remains challenging [37]. Currently, the best way to identify cell death *in vivo* relies on measuring the amounts of intracellular molecules released by dying cells [38]; however many of these markers are not unique to cell death, and their cellular sources cannot be defined [39], making this assessment at the moment equivocal.

Determining Plac8’s direct impact on Th1-driven inflammation has proven to be complex; however, these studies demonstrate that Plac8 has potential to serve as a viable therapeutic target. Through our CD4 T cell experiments, we have shown that Plac8 can limit IFNγ production by CD4 T cells, suggesting that Plac8 could be utilized to limit cellular inflammation and provide a longer-lasting, more efficacious alternative to the current anti-inflammatory treatments. In addition, we determined that global Plac8 expression promotes the establishment of memory CD8 T cells following influenza infection, which may promote efficacy rates in the current influenza vaccines. Although the precise function of Plac8 during Th1-driven inflammatory responses remains elusive, for the first time, these data show that Plac8 serves an immunoregulatory role during Th1 type immune responses and should be further explored for potential therapeutic benefit.

## Supporting information

S1 Fig Plac8 ablation does not alter T cell development. Naïve WT and *Plac8*^*-/-*^ mice were sacrificed, and the thymi and spleens were harvested. To compare the development of thymic T cells, thymocytes first gated on CD90.2^+^ before being distinguished by CD4 vs CD8 expression (**A**). CD4-CD8- (DN) thymocytes were further characterized as DN1-DN4 populations according to CD44 and CD25 expression. The frequencies of each thymocyte population were then plotted as a bar graph. To compare the T cell compartments between naïve WT and *Plac8*^*-/-*^ mice, splenocytes were gated as either CD4^+^, CD8^-^ to designate the CD4 T cell population or CD4^-^, CD8^+^ to designate the CD8 T cell population (**B**). The frequency and total number of CD4 and CD8 T cells for the WT and *Plac8*^*-/-*^ were determined. WT (n = 4) and *Plac8*^*-/-*^ (n = 3). P values were determined using unpaired Student’s t test. * (P < 0.05).
(TIF)

S2 Fig Efficiency of CD4 Th cell differentiation *in vitro*. Naïve CD4 T cells were TCR-activated by α-CD3 and α-CD3 and differentiated in the presence of IL-12 (Th1 conditions), IL-4 (Th2 conditions), TFG-β and IL-6 (Th17 conditions), TGF-β and IL-2 (iTreg conditions) or media alone (Th0) for 3d. Relative expression of the indicated Th subset signature cytokine or transcription factor were determined by RT-qPCR using Th0 cells as the baseline expression level. Data are representative of three independent cohorts. *P* values were determined by one-way ANOVA * (*P* < 0.05), *** (*P* < 0.001).
(TIF)

S3 Fig Plac8 does not affect inflammatory cytokine production by CD4 T cells after *Citrobacter rodentium* infection. C57BL/6 CD45.2/.1 heterozygote hosts were irradiated with a single dose of 1,100 rad and reconstituted with 3 million WT (CD45.1) and 3 million *Plac8*^*-/-*^ (CD45.2) bone marrow cells. Two months after immune cell reconstitution, hosts were infected with 1 x 10^9^ to 3 x 10^9^ CFU of a luminescent strain of *Citrobacter rodentium* ICC180 (kindly provided by Gad Frankel at Imperial College, London, United Kingdom), via gastric gavage in a total volume of 200μl. The dose was confirmed through retrospective plating on LB agar plates. Three days post gastric gavage, mice were anesthetized using isoflurane and imaged for 30 seconds using an IVIS Lumina imager (PerkinElmer) to confirm infection status. 14 dpi, lymphocytes were isolated from the lamina propria, spleen, mesenteric lymph nodes and resuspended to 1x10^6^ cells/mL before *ex vivo* stimulation with 50 ng/mL PMA, 0.5 μg/mL ionomycin, and Golgi transport inhibitor according to the manufacturer’s directions (BD Biosciences) for 4 h at 37°C. Cells were then surface stained for CD45.1 (A20), CD45.2 (104), CD4 (RM4-5), and TCRβ (H57-597) at 4°C for 20 min before being intracellularly stained for IFNγ (XMG1.2), TNF (MP6-XT22), and IL-17A (eBio1787) as described in materials and methods. Lines between black (WT) and white (KO) circles represent a single host. This experiment contains 5 mice and is a representative of 3 independent experiments.
(TIF)

S4 Fig Plac8 ablation does not alter effector CD8 T cell IFNγ secretion nor differentiation during influenza infection. CD45.1/.2 heterozygous mice were given an adoptive transfer of 1,000 WT OT-I T cells and 1,000 *Plac8*^*-/-*^ OT-I T cells one day prior to X31-OVA infection. 8 dpi, mice were sacrificed and lungs, mdLNs, and spleens were processed for flow cytometry. Before staining, each sample was split into two for separate staining panels. Half of the samples were stimulated with SIINFEKL OVA-peptide for 5h at 37°C or with media alone as a negative control. After stimulation, cells were surface stained before being fixed and permeabilized for intracellular staining. Cells were gated as Vα2^+^ and CD8^+^ before being designated as CD45.1^+^ (WT) or CD45.2^+^ (KO). Once the WT and *Plac8*^*-/-*^ OT-I cells were identified, the frequency of IFNγ^+^ cells was determined as seen in the representative lung tissue (**A**). This was performed for the lung, mdLN, and spleen (**B**). A seperate second staining panel was performed to identify phenotypic markers associated with effector CD8 T cell fate. Short-lived effector cells (SLECs) express KLRG1 and are predicted to undergo apoptosis during contraction. Memory precursor effector cells (MPECs) express CD127 and are predicted to become memory CD8 T cells after infection. Early effector cells (EECs) express neither of these phenotypic markers and have the potential to become SLECs or MPECs as the infection progresses. WT and *Plac8*^*-/-*^ OT-I cells were distinguished based upon CD45 expression and SLECs, MPECs, and EECs were identified by KLRG1 and CD127 expression using a quadrant gating strategy (**C**). This was performed for lungs and mdLN and cumulative data shown (**D**). This experiment has an n = 5 of each genotype and is representative of two independent experiments. *P* values were determined using paired Student’s t test.
(TIF)
