# Supplementary figures and images for: Placenta-specific 8 limits IFNγ production by CD4 T cells in vitro and promotes establishment of influenza-specific CD8 T cells in vivo
Source: PLoS One. 2020 Jul 8;15(7):e0235706. doi: 10.1371/journal.pone.0235706 (PMC7343148; doi:10.1371/journal.pone.0235706)

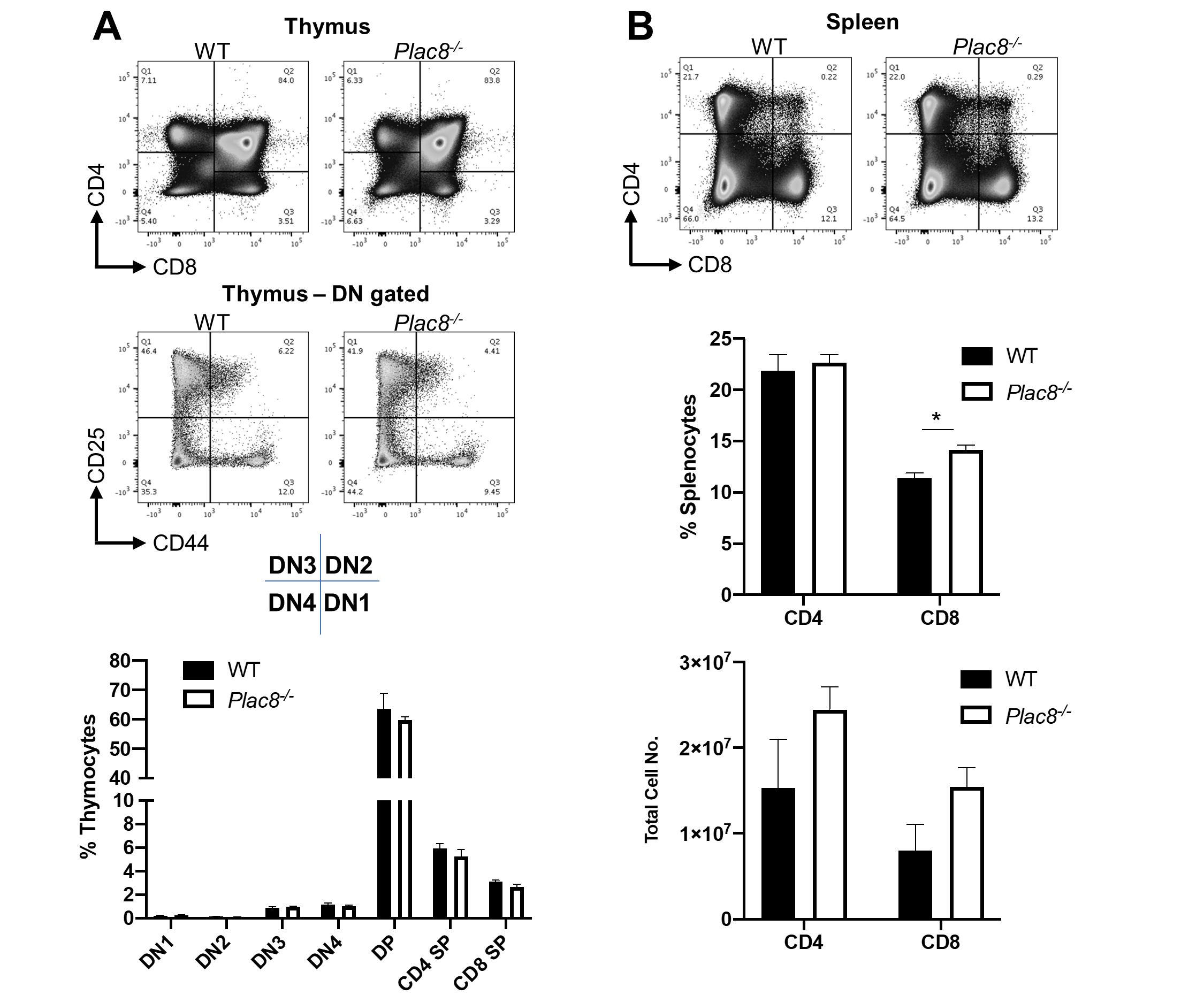

Supplement: S1 Fig — Naïve WT and Plac8-/- mice were sacrificed, and the thymi and spleens were harvested. To compare the development of thymic T cells, thymocytes first gated on CD90.2+ before being distinguished by CD4 vs CD8 expression (A). CD4-CD8- (DN) thymocytes were further characterized as DN1-DN4 populations according to CD44 and CD25 expression. The frequencies of each thymocyte population were then plotted as a bar graph. To compare the T cell compartments between naïve WT and Plac8-/- mice, splenocytes were gated as either CD4+, CD8- to designate the CD4 T cell population or CD4-, CD8+ to designate the CD8 T cell population (B). The frequency and total number of CD4 and CD8 T cells for the WT and Plac8-/- were determined. WT (n = 4) and Plac8-/- (n = 3). P values were determined using unpaired Student’s t test. * (P < 0.05). (TIF) [file pone.0235706.s001.tif]

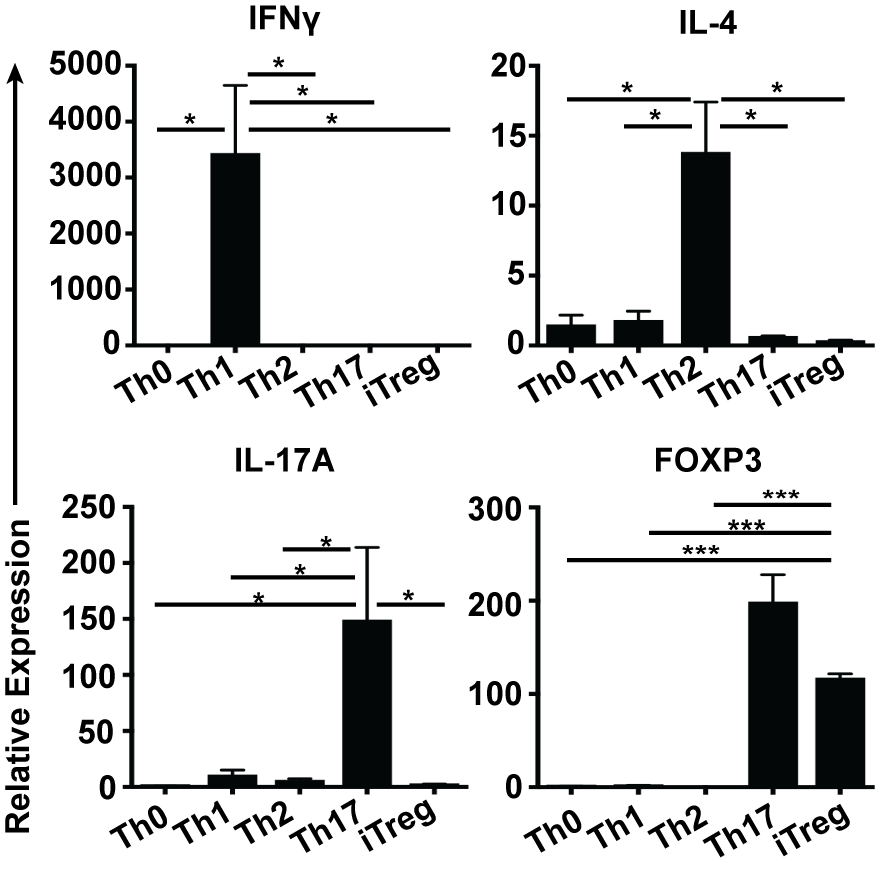

Supplement: S2 Fig — Naïve CD4 T cells were TCR-activated by α-CD3 and α-CD3 and differentiated in the presence of IL-12 (Th1 conditions), IL-4 (Th2 conditions), TFG-β and IL-6 (Th17 conditions), TGF-β and IL-2 (iTreg conditions) or media alone (Th0) for 3d. Relative expression of the indicated Th subset signature cytokine or transcription factor were determined by RT-qPCR using Th0 cells as the baseline expression level. Data are representative of three independent cohorts. P values were determined by one-way ANOVA * (P < 0.05), *** (P < 0.001). (TIF) [file pone.0235706.s002.tif]

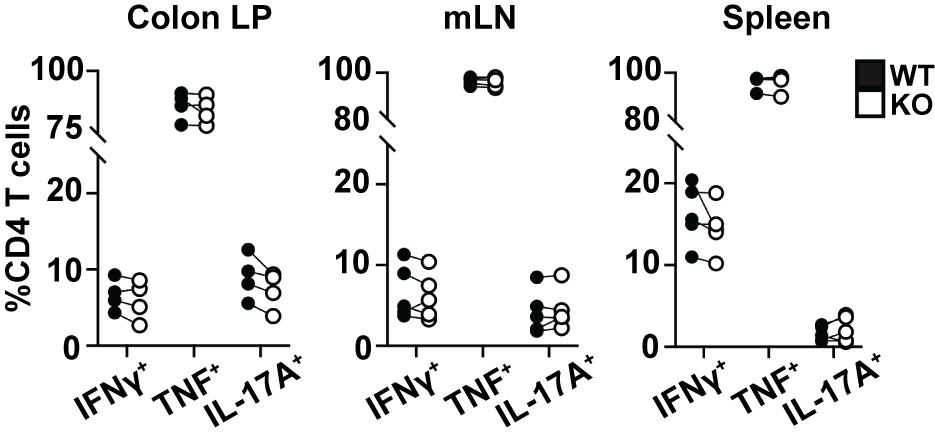

Supplement: S3 Fig — C57BL/6 CD45.2/.1 heterozygote hosts were irradiated with a single dose of 1,100 rad and reconstituted with 3 million WT (CD45.1) and 3 million Plac8-/- (CD45.2) bone marrow cells. Two months after immune cell reconstitution, hosts were infected with 1 x 10^9^ to 3 x 10^9^ CFU of a luminescent strain of Citrobacter rodentium ICC180 (kindly provided by Gad Frankel at Imperial College, London, United Kingdom), via gastric gavage in a total volume of 200μl. The dose was confirmed through retrospective plating on LB agar plates. Three days post gastric gavage, mice were anesthetized using isoflurane and imaged for 30 seconds using an IVIS Lumina imager (PerkinElmer) to confirm infection status. 14 dpi, lymphocytes were isolated from the lamina propria, spleen, mesenteric lymph nodes and resuspended to 1x10^6^ cells/mL before ex vivo stimulation with 50 ng/mL PMA, 0.5 μg/mL ionomycin, and Golgi transport inhibitor according to the manufacturer’s directions (BD Biosciences) for 4 h at 37°C. Cells were then surface stained for CD45.1 (A20), CD45.2 (104), CD4 (RM4-5), and TCRβ (H57-597) at 4°C for 20 min before being intracellularly stained for IFNγ (XMG1.2), TNF (MP6-XT22), and IL-17A (eBio1787) as described in materials and methods. Lines between black (WT) and white (KO) circles represent a single host. This experiment contains 5 mice and is a representative of 3 independent experiments. (TIF) [file pone.0235706.s003.tif]

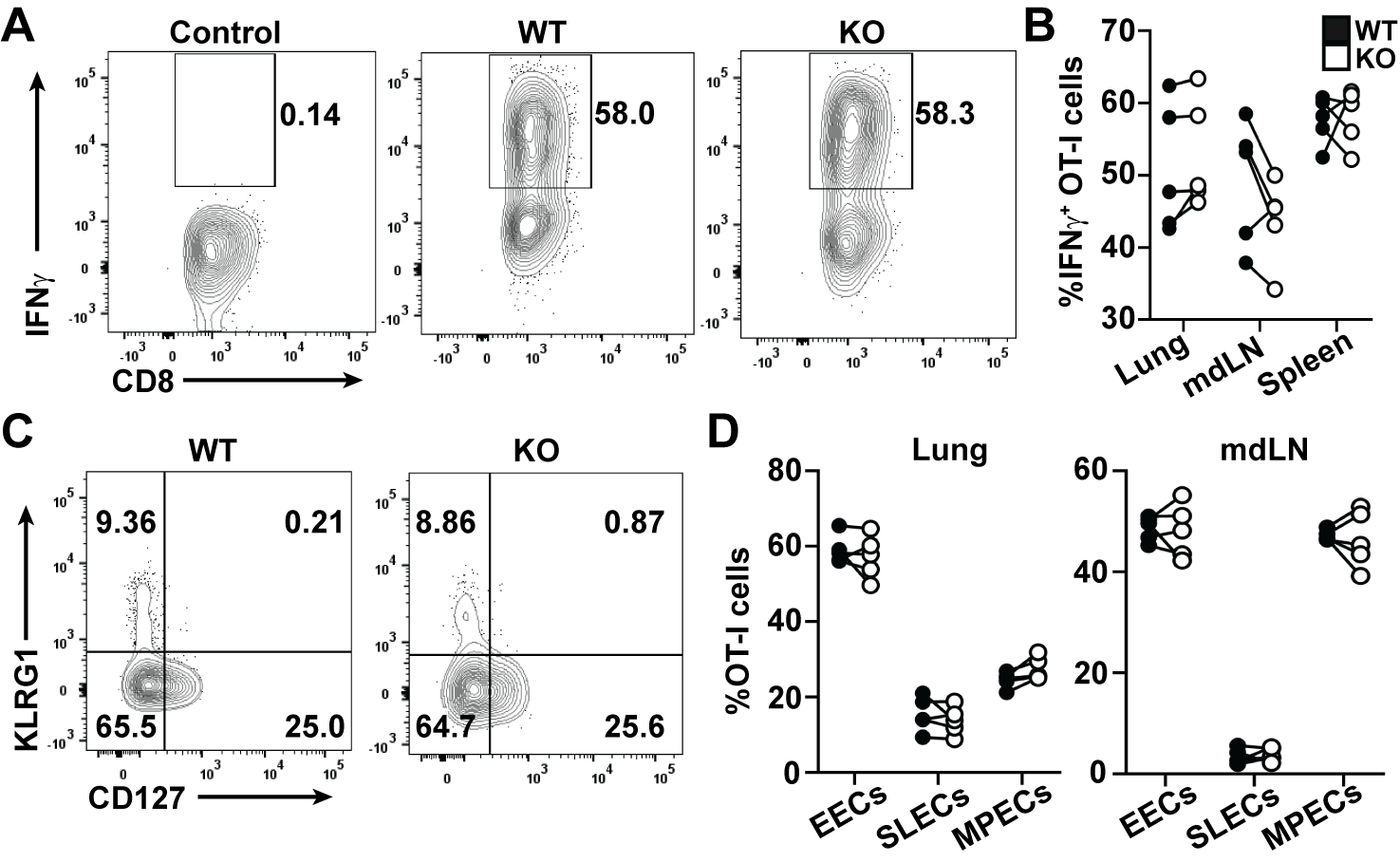

Supplement: S4 Fig — CD45.1/.2 heterozygous mice were given an adoptive transfer of 1,000 WT OT-I T cells and 1,000 Plac8-/- OT-I T cells one day prior to X31-OVA infection. 8 dpi, mice were sacrificed and lungs, mdLNs, and spleens were processed for flow cytometry. Before staining, each sample was split into two for separate staining panels. Half of the samples were stimulated with SIINFEKL OVA-peptide for 5h at 37°C or with media alone as a negative control. After stimulation, cells were surface stained before being fixed and permeabilized for intracellular staining. Cells were gated as Vα2+ and CD8+ before being designated as CD45.1+ (WT) or CD45.2+ (KO). Once the WT and Plac8-/- OT-I cells were identified, the frequency of IFNγ+ cells was determined as seen in the representative lung tissue (A). This was performed for the lung, mdLN, and spleen (B). A seperate second staining panel was performed to identify phenotypic markers associated with effector CD8 T cell fate. Short-lived effector cells (SLECs) express KLRG1 and are predicted to undergo apoptosis during contraction. Memory precursor effector cells (MPECs) express CD127 and are predicted to become memory CD8 T cells after infection. Early effector cells (EECs) express neither of these phenotypic markers and have the potential to become SLECs or MPECs as the infection progresses. WT and Plac8-/- OT-I cells were distinguished based upon CD45 expression and SLECs, MPECs, and EECs were identified by KLRG1 and CD127 expression using a quadrant gating strategy (C). This was performed for lungs and mdLN and cumulative data shown (D). This experiment has an n = 5 of each genotype and is representative of two independent experiments. P values were determined using paired Student’s t test. (TIF) [file pone.0235706.s004.tif]
